# A systematic review and meta-analysis of the prevalence of hepatitis B virus infection among pregnant women in Nigeria

**DOI:** 10.1371/journal.pone.0259218

**Published:** 2021-10-29

**Authors:** Babayemi O. Olakunde, Daniel A. Adeyinka, Olubunmi A. Olakunde, Olalekan A. Uthman, Florence O. Bada, Yvonne A. Nartey, Dorcas Obiri-Yeboah, Elijah Paintsil, Echezona E. Ezeanolue

**Affiliations:** 1 Department of Community Prevention and Care Services, National Agency for the Control of AIDS, Abuja, Nigeria; 2 Center for Translation and Implementation Research, University of Nigeria, Nsukka, Enugu, Nigeria; 3 Department of Public Health, National AIDS and STI Control Programme, Federal Ministry of Health, Abuja, Nigeria; 4 Department of Community Health and Epidemiology, College of Medicine, University of Saskatchewan, Saskatoon, Canada; 5 Department of Disease Control and Immunization, Ondo State Primary Health Care Development Agency, Ondo, Nigeria; 6 Warwick Centre for Global Health, Division of Health Sciences, University of Warwick Medical School, Coventry, United Kingdom; 7 Division of Epidemiology and Biostatistics, Department of Global Health, Faculty of Health Sciences, Stellenbosch University, Cape Town, South Africa; 8 Department of Epidemiology and Public Health, University of Maryland School of Medicine, Baltimore, Maryland, United States of America; 9 International Research Center of Excellence, Institute of Human Virology Nigeria, Abuja, Nigeria; 10 Department of Medicine, Cape Coast Teaching Hospital, Cape Coast, Ghana; 11 Department of Medical Epidemiology and Biostatistics, Karolinska Institute, Stockholm, Sweden; 12 Department of Microbiology and Immunology, University of Cape Coast, Cape Coast, Ghana; 13 Department of Pediatrics, Yale School of Medicine, New Haven, Connecticut, United States of America; 14 Department of Epidemiology of Microbial Diseases, Yale School of Public Health, New Haven, Connecticut, United States of America; 15 Department of Pharmacology, Yale School of Medicine, New Haven, Connecticut, United States of America; 16 Healthy Sunrise Foundation, Las Vegas, Nevada, United States of America; University of Cape Town, SOUTH AFRICA

## Abstract

**Background:**

Nigeria has a high burden of hepatitis B virus (HBV) infection, commonly acquired through vertical transmission. However, there is a lack of an efficient surveillance system for monitoring and understanding the epidemiology of HBV among pregnant women. Building on a previous review on the prevalence of HBV in Nigeria (2000–2013), we conducted a systematic review and meta-analysis of HBV prevalence among pregnant women in Nigeria.

**Methods:**

Four electronic databases PubMed, Embase, Global Health, and Scopus were systematically searched from January 2014 to February 2021. We also searched the African Journal Online and manually scanned the reference lists of the identified studies for potentially eligible articles. Observational studies that reported the prevalence of HBsAg and/or HBeAg among pregnant women in peer-reviewed journals were included in the study. We performed a meta-analysis using a random-effects model. We defined HBV infection as a positive test to HBsAg.

**Results:**

From the 158 studies identified, 20 studies with a total sample size of 26, 548 were included in the meta-analysis. The pooled prevalence of HBV infection among pregnant women across the studies was 6.49% (95% confidence interval [CI] = 4.75–8.46%; I^2^ = 96.7%, p = 0.001; n = 20). The prevalence of HBV was significantly lower among pregnant women with at least secondary education compared with those with no education or primary education (prevalence ratio = 0.7, 95% CI = 0.58–0.87; n = 10). However, the prevalence of HBV was not significantly different by age, religion, marital status, or tribe. The prevalence of HBV was not significantly different among pregnant women with previous surgery, blood transfusion, multiple lifetime sex partners, tribal marks, tattoos, scarification, or sexually transmitted infections, compared with those without these risk factors. From a total sample size of 128 (n = 7), the pooled prevalence of HBeAg among HBV-infected pregnant women was 14.59% (95% CI = 4.58–27.99%; I^2^ = 65.5%, p = 0.01). Subgroup analyses of HBV infection by study region and screening method, and meta-regression analysis of the study year, sample size, and quality rating were not statistically significant.

**Conclusions:**

There is an intermediate endemicity of HBV infection among pregnant women in Nigeria. Interventions, such as routine antenatal HBV screening, antiviral prophylaxis for eligible pregnant women, and infant HBV vaccination should be scaled up for the prevention of perinatal transmission of HBV infection in Nigeria.

## Background

With over 20 million people estimated to be infected with Hepatitis B virus (HBV) infection, Nigeria has the largest number of people living with HBV infection in sub-Saharan Africa (SSA) and ranks third after China and India, globally [1]. The 2018 Nigeria HIV/AIDS Impact and Survey reported the prevalence of HBV among persons aged 15–49 years is 8.6%, with the prevalence among males (11.1%) about twice that of females (6.1%) [2]. In Nigeria, HBV is the most common cause of liver cancer [3] and the fourth leading cause of cancer deaths [4]. Nonetheless, it has continued to be a silent epidemic, as most of the people infected are undiagnosed and do not access treatment and prevention services [1, 5].

In highly endemic countries in SSA, HBV is commonly acquired through perinatal transmission from HBV-infected mothers [6, 7], particularly those who have a high viral load and/or are positive for the hepatitis B e antigen (HBeAg) [8–11]. Approximately 370,000 newborns are perinatally infected with HBV in SSA annually [12]. While HBV infection in adulthood leads to chronic hepatitis in less than 5% of adults, about 80–90% of persons infected in the first year of life develop chronic hepatitis [13, 14]. However, perinatal transmission of HBV is preventable with safe and effective vaccines [15–17]. Hepatitis B vaccine birth-dose (HepB-BD) reduces the risk of perinatal transmission to 20–30% in infants of hepatitis B e antigen (HBeAg)-positive mothers and less than 0.5% in those born to HBeAg-negative mothers [12]. Where available, administering hepatitis B immune globulin (HBIG) to the infants and maternal antiviral therapy can be of additional benefit, particularly if mothers are HBeAg-positive [12, 18–20]. The effectiveness of HBV vaccines to prevent transmission underpins the current efforts to eliminate HBV infection as a public health threat by 2030 globally [21]. In 2016, Nigeria developed a 5-year strategic plan (2016–2020) as a road map to eliminating viral hepatitis by 2030 [22].‬‬‬‬‬‬‬‬‬‬‬‬‬‬‬‬‬‬‬‬‬‬‬‬‬‬‬‬‬‬‬‬‬‬‬‬‬‬‬‬‬‬‬‬‬‬‬‬‬‬‬‬‬‬‬‬‬‬‬‬‬‬‬‬‬‬‬‬‬‬‬‬‬‬‬‬‬‬‬‬‬‬‬‬‬‬‬‬‬‬‬‬‬‬‬‬‬‬‬‬

Despite the high burden of HBV in Nigeria, there is no efficient surveillance system for monitoring and understanding the epidemiology of the infection [22, 23]‬‬‬‬‬‬‬‬‬‬‬‬‬‬‬‬‬‬‬‬‬‬‬‬‬‬‬‬‬‬‬‬‬‬‬‬‬‬‬‬‬‬‬‬‬‬‬‬‬‬‬‬‬‬‬‬‬‬‬‬‬‬‬‬‬‬‬‬‬‬‬‬‬‬‬‬‬‬‬‬‬‬‬‬‬‬‬‬‬‬‬‬‬‬‬‬‬‬‬‬. Program data are deficient as pregnant women are not routinely screened for HBV [24, 25] and population-based serosurveys are not regularly conducted [22, 26, 27]. Accurate estimates of the burden of HBV in Nigeria, especially among pregnant women, are needed for rational planning of health services and would allow public-health policymakers to assign sufficient priority and resources to its management and prevention. In the absence of surveillance data, information from multiple studies has been used to generate prevalence estimates. In 2014, a meta-analysis of studies published between 2000 and 2013 in Nigeria estimated the HBV prevalence in pregnant women as 14.1% (95% confidence interval [CI] = 9.6, 18.6%) from 14 studies [26]. However, the review focused on women attending antenatal care in health facilities. Furthermore, the review did not consider the prevalence of HBV by sociodemographic characteristics, the risk factors associated with HBV, and the prevalence of HBeAg among HBV-infected pregnant women. Similar limitations were present in the 2018 national household survey among a population of 435 pregnant women in Nigeria [2].

Since the systematic review and meta-analysis on the prevalence of HBV, including pregnant women, in Nigeria, was published seven years ago [26], several studies have become available. Estimates from these studies may differ from older studies, considering the recent efforts to eliminate perinatal HBV transmission [22].‬‬‬‬‬‬‬‬‬‬‬‬‬‬‬‬‬‬‬‬‬‬‬‬‬‬‬‬‬‬‬‬‬‬‬‬‬‬‬‬‬‬‬‬‬‬‬‬‬‬‬‬‬‬‬‬‬‬‬‬‬‬‬‬‬‬‬‬‬‬‬‬‬‬‬‬‬‬‬‬‬‬‬‬‬‬‬‬‬‬‬‬‬‬‬‬‬‬‬‬ Accordingly, this systematic review aimed to provide expanded up-to-date evidence on the epidemiology of HBV among pregnant women in Nigeria. The objectives were to determine: (i) the prevalence of HBV infection among pregnant women, (ii) the differences in the prevalence of HBV by sociodemographic characteristics and by known risk factors associated with HBV infection among pregnant women, and (iii) the prevalence of HBeAg among HBV-infected pregnant women in Nigeria.

## Methods

### Design

This study was performed and reported using the guidelines for Preferred Reporting Items for Systematic Reviews and Meta-Analyses (PRISMA) [28]. The protocol for this review was guided by previous reviews and meta-analyses on the epidemiology of HBV among pregnant women [29–31].

### Search strategy

We systematically searched PubMed, Embase, Global Health, and Scopus for eligible articles published between January 1, 2014 and February 4, 2021. Our search terms included keywords relating to: “Hepatitis B”, “Pregnancy”, and “Nigeria” (see S1 File for search strategy for PubMed). We also searched the African Journal Online and manually scanned reference lists of the identified studies for potentially eligible articles. We restricted our search to studies published in English language. We defined HBV infection as a positive test result to hepatitis B surface antigen (HBsAg) based on a rapid diagnostic test (RDT), enzyme-linked immunosorbent assay (ELISA), or both. The sociodemographic characteristics considered included: age (young pregnant women (<25 years) vs older pregnant women (≥25 years)), educational attainment (none or primary education vs secondary or higher education), monthly income (below minimum wage (<₦30,0000) vs minimum wage or above (≥₦30,000)), religion (Christianity vs Islam), and any other sociodemographic characteristics reported in at least two papers. The known risk factors for HBV considered were previous surgery, blood transfusion, scarification, tribal marks, multiple sex partners, and any other risk factors reported in at least two papers.

### Inclusion and exclusion criteria

We considered both experimental and observational quantitative research studies published in peer-reviewed journals. Articles were eligible to be included in this study if they were conducted in Nigeria, screened pregnant women for HBV, reported the prevalence of HBsAg and/or HBeAg among HBsAg-positive women, and/or the prevalence of HBsAg by sociodemographic characteristics or known risk factors associated with HBV infection. We excluded studies that did not include pregnant women or did not disaggregate data for pregnant women. Studies were also excluded if the diagnosis of HBV infection was not based on HBsAg positive test or not described. Studies that used the same data were also excluded, retaining the one with more information regarding the inclusion criteria. We excluded studies deemed to be published in questionable, scholarly open-access (predatory) journals, using a guide by Ross-White and colleagues [32].

### Study selection and abstraction

The study selection was conducted in phases based on the inclusion and exclusion criteria. Two authors (BOO and DAA) independently screened the titles and abstracts of the articles. The full articles of those deemed eligible were retrieved and independently screened by the two authors. At each phase of the screening, we ensured there was an agreement between the two authors on the selected articles, and cases of conflict were resolved by a third author (OAO). The data from the included studies were extracted using a pretested tool developed by the authors (S2 File). Two authors (BOO and OAO) retrieved information including, the first author’s surname, publication year, study location, study design, study year, the HBV-specific antigen reported, screening method, number of pregnant women screened for HBV (HBsAg), number of screened pregnant women who tested positive for HBV (HBsAg), HBV status by sociodemographic characteristics, the number of HBV-infected women who tested positive for HBeAg, and the reported risk factors. Where required, authors of the included studies were contacted for additional information. Two other authors (DAA and EEE) randomly selected and cross-checked the extracted data.

### Quality assessment

The quality of the papers included in the study was assessed by two authors (BOO and OAO) using the Joanna Briggs Institute Critical Appraisal Checklist for Studies Reporting Prevalence Data [33]. The checklist assesses the methodological quality of prevalence studies based on nine questions (S2 File). Possible responses were ‘yes’; ‘no’; ‘unclear’; or ‘not applicable’. We assigned a maximum score of 1 to each question, with a potential minimum score of 0 and a maximum of 9. However, it was decided a priori not to exclude any study based on the quality rating.

### Analysis

We pooled the prevalence of HBV in the studies using the procedure for binomial data [34]. The prevalence of HBV by sociodemographic and known risk factors were compared using relative risk, referred to as prevalence ratio (PR) in this study. The HBV prevalence and the PR were estimated using a random-effects meta-analysis model with Freeman-Tukey double arcsine transformation [35] and DerSimonian and Laird method [36], respectively. Statistical heterogeneity was assessed by the Cochran’s Q statistic, with p-value <0.1 as the level of statistical significance [37]. It was further assessed with I^2^ statistic which shows the percentage of the variability in pooled estimates that is due to heterogeneity rather than chance [37]. We considered I^2^ statistic values of 50% or more as substantial heterogeneity [38]. For HBV prevalence, subgroup analyses were performed to identify the possible sources of heterogeneity and also for group comparison. The studies were grouped by study region and screening methods. Meta-regression was also performed to assess the effect of the study sample size, year of study, and quality rating [39]. Publication bias was assessed visually with a funnel plot and using Egger tests, with a p-value <0.05 considered statistically significant [40]. The meta-analysis was conducted using STATA V.16.0 for Windows (Stata Statistical Software: Release 16. College Station, TX: StataCorp LLC).

## Results

### Search results

**Fig 1:**
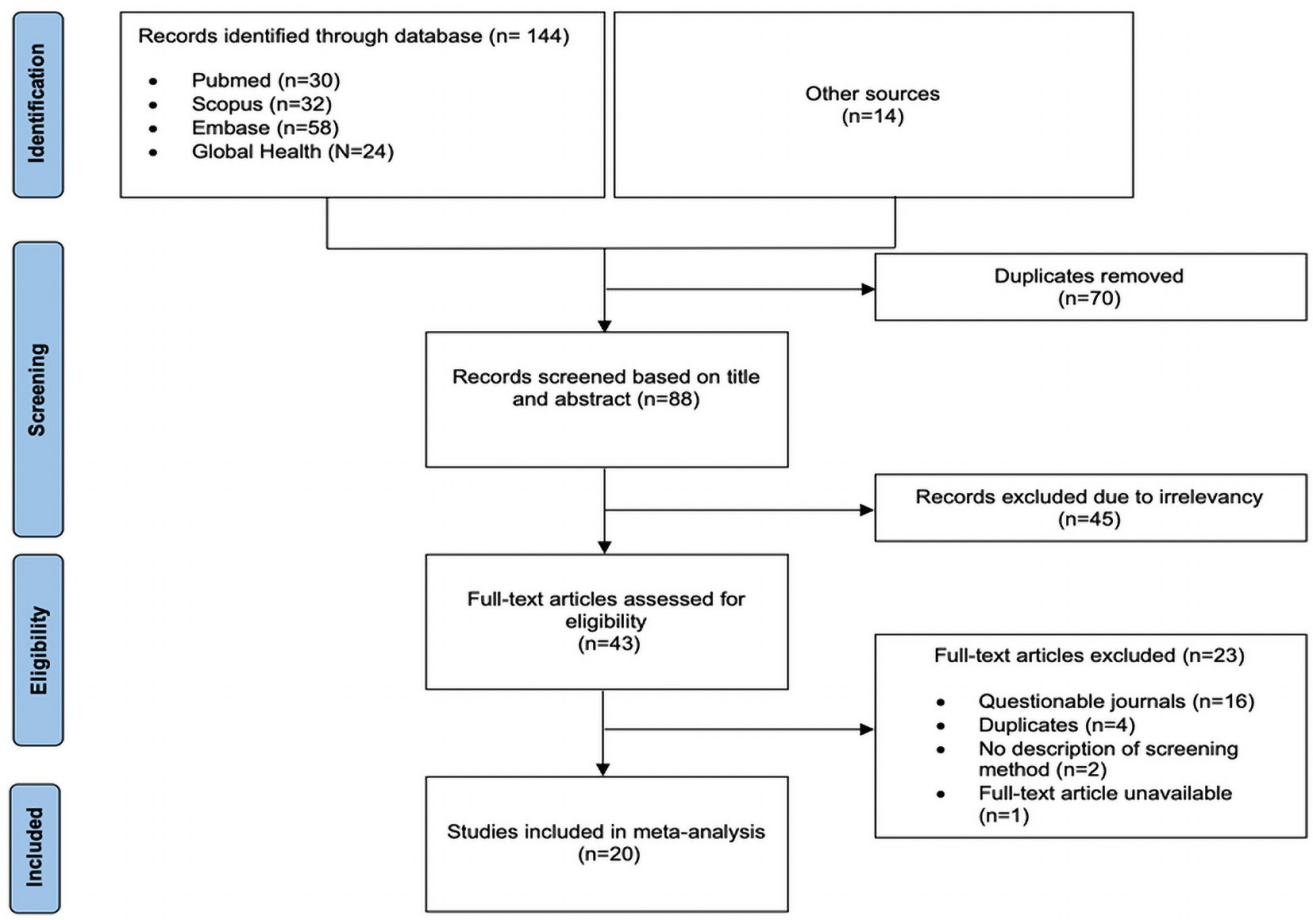
PRISMA flow diagram of the process of study identification and selection.

Fig 1 shows the PRISMA flow diagram for study selection. A total of 144 studies were identified through the four databases with an additional 14 identified from other sources. After the removal of duplicates, the titles and abstracts of 88 articles were screened, out of which 45 were found irrelevant. The full-text articles of 43 studies were retrieved and assessed for eligibility. Twenty articles were included in the meta-analysis and 23 articles were excluded with reasons illustrated in Fig 1.

The characteristics of the included studies are summarized in Table 1. The publication year of the included studies ranged from 2014 to 2021. The studies included had a total sample size of 26, 548. Seventeen of the 20 studies (85%) were facility-based cross-sectional studies [41–57], two studies were retrospective chart reviews [58, 59], and one was a community-based cross-sectional study [60]. Half of the studies were conducted in the Northern region (North Central = 5; North East = 2; and North West = 3) [41–44, 46, 52, 54, 56, 57, 60] and the remaining 50% were conducted in the Southern region (South East = 1 and South West = 9) [45, 47–50, 53, 55, 58, 59]. HBsAg status was reported in all of the 20 studies, however, only seven studies [41, 42, 50, 52–55] reported both HBsAg and HBeAg status. HBsAg test was performed with a rapid diagnostic test (RDT) in eight studies [44, 46, 47, 49, 54, 57, 59, 60], while enzyme-linked immunosorbent assay (ELISA) was used in six studies [43, 45, 51, 53, 55, 58]. Six studies used RDT as the initial test and ELISA as the confirmatory test [41, 42, 48, 50, 52, 56]. ELISA was used to test for HBeAg in all the seven studies that assessed it [41, 42, 50, 52–55]. The methodological quality score was ≥ 7 in eight studies [43, 46, 50–52, 57, 58, 60], while the other twelve studies scored between 4 and 6 [41, 42, 44, 45, 47–49, 53–56, 59].

**Table 1:** Summary characteristics of studies included in the review, 2014–2021.

First author, publication year	Study year	Study type	Sample size	Study region	Study zone (state)	Screening method	Test conducted	Quality rating
Aba, 2016 [41]	2011	Facility-based cross-sectional survey	800	North	North West (Kaduna)	RDT and ELISA	HBsAg and HBeAg	6
Abulude, 2017 [42]	2016	Facility-based cross-sectional survey	160	North	North West (Kano)	RDT and ELISA	HBsAg and HBeAg	4
Adegbesan-Omilabu, 2015 [50]	2014	Facility-based cross-sectional survey	150	South	South West (Lagos)	RDT and ELISA	HBsAg and HBeAg	7
Adeogun, 2020 [48]	NS	Facility-based cross-sectional survey	2998	South	South West (Ondo)	RDT and ELISA	HBsAg	4
Adeyemi, 2014 [51]	2011	Facility-based cross-sectional survey	628	South	South West (Oyo)	ELISA	HBsAg	7
Aluor, 2016 [52]	2012	Facility-based cross-sectional survey	310	North	North Central (Benue)	RDT and ELISA	HBsAg and HBeAg	7
Anaedobe, 2015 [53]	2013	Facility-based cross-sectional survey	180	South	South West (Oyo)	ELISA	HBsAg and HBeAg	6
Erhabor, 2020 [54]	2015	Facility-based cross-sectional survey	117	North	North West (Sokoto)	RDT	HBsAg and HBeAg	6
Ifeorah, 2017 [55]	2012	Facility-based cross-sectional survey	272	South	South West (Oyo)	ELISA	HBsAg and HBeAg	4
Ikeako, 2014 [58]	2006	Retrospective chart review	1239	South	South East (Enugu)	ELISA	HBsAg	7
Jibrin, 2016 [56]	2012	Facility-based cross-sectional survey	2462	North	North East (Bauchi)	RDT and ELISA	HBsAg	5
Magaji, 2021 [57]	2017	Facility-based cross-sectional survey	3238	North	North Central (Plateau)	RDT	HBsAg	7
Mustapha, 2020 [43]	2018	Facility-based cross-sectional survey	210	North	North East (Bauchi)	ELISA	HBsAg	8
Nongo, 2016 [44]	2012	Facility-based cross-sectional survey	200	North	North Central (FCT)	RDT	HBsAg	4
Ojo, 2016 [59]	2013	Retrospective chart review	373	South	South West (Ondo)	RDT	HBsAg	5
Oluremi, 2020 [45]	2019	Facility-based cross-sectional survey	904	South	South West (Oyo)	ELISA	HBsAg	5
Omatola, 2019 [46]	2017	Facility-based cross-sectional survey	200	North	North Central (Kogi)	RDT	HBsAg	7
Opaleye, 2016 [47]	2014	Facility-based cross-sectional survey	182	South	South West (Osun)	RDT	HBsAg	5
Osho, 2019 [49]	2015	Facility-based cross-sectional survey	1758	South	South West (Ondo)	RDT	HBsAg	5
Talla, 2021 [60]	2017	Community-based cross-sectional survey	10167	North	North Central (Benue)	RDT	HBsAg	8

ELISA: Enzyme-linked immunosorbent assay; RDT: Rapid diagnostic test; HBsAg: Hepatitis B surface antigen; HBeAg: Hepatitis B e antigen; NS: Not stated

### HBV prevalence

**Fig 2:**
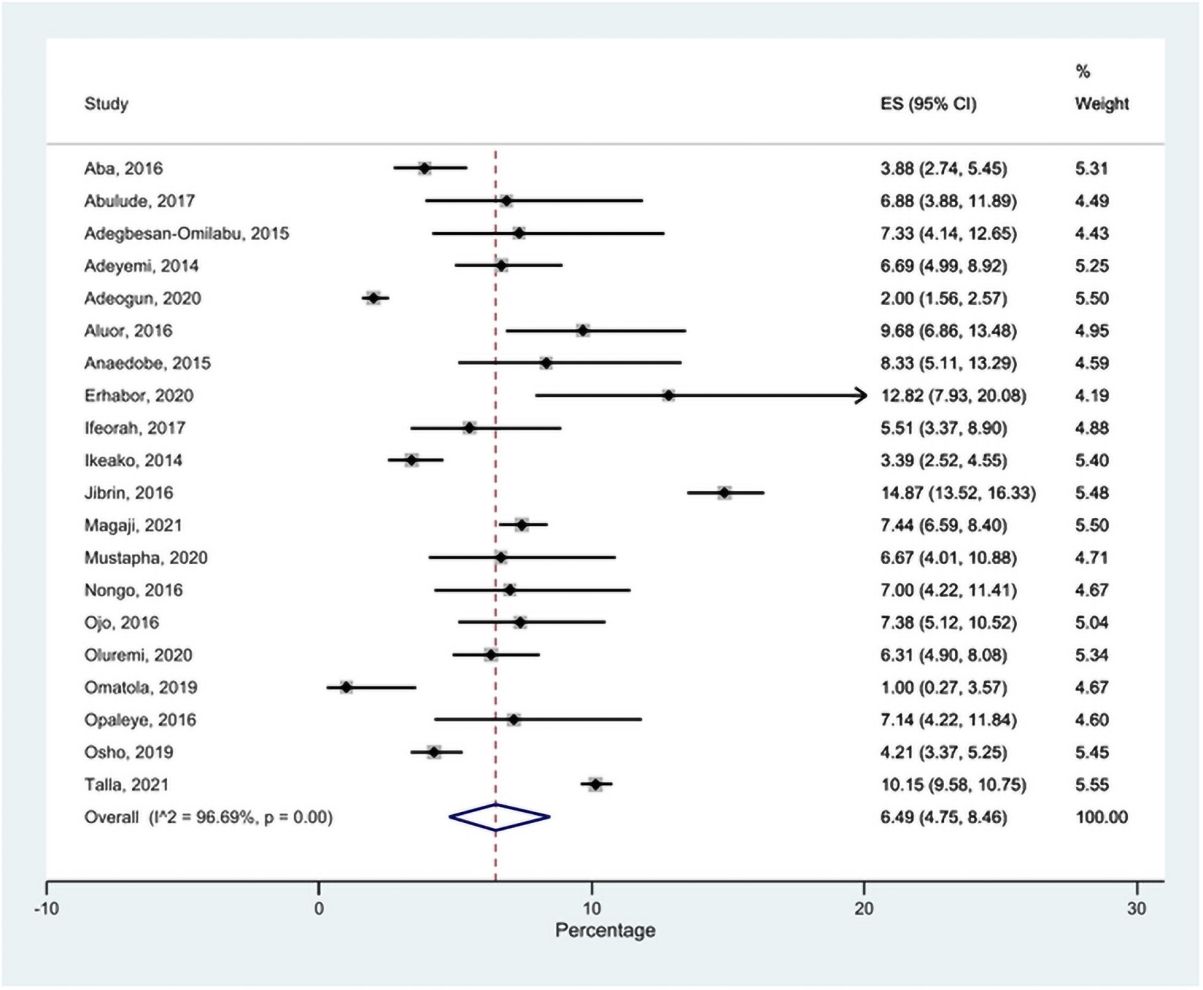
Forest plot of HBV prevalence among pregnant women in Nigeria, 2014–2021.

The HBV prevalence in the 20 studies included in the meta-analysis ranged from 1.00% to 14.87% (Fig 2). Out of the 20 studies, only five studies reported a prevalence of more than 8%. The pooled prevalence of HBV among pregnant women in the 20 studies was 6.49% (95% confidence interval [CI] = 4.75–8.46%; I^2^ = 96.7%, p = 0.001) (Fig 2).

### HBV prevalence and prevalence ratios by sociodemographic characteristics and risk factors

The HBV prevalence varied by sociodemographic characteristics and known risk factors (Table 2). The results indicated a significantly lower prevalence of HBV in pregnant women who had at least secondary education compared with those who had primary or no education (PR = 0.71, 95% CI = 0.58–0.87). However, the prevalence of HBV was not significantly different by age, religion, marital status, or tribe (Table 2). Similarly, the prevalence of HBV was not significantly different among pregnant women with previous surgery (PR = 1.08, 95% CI = 0.90–1.29), blood transfusion (PR = 1.19, 95% CI = 0.95–1.48), multiple lifetime sex partners (PR = 0.80, 95% CI = 0.35–1.82), tattoos (PR = 1.02, 95% CI = 0.72–1.45), tribal marks (PR = 0.19, 95% CI = 0.02–1.45), scarification (PR = 0.87, 95% CI = 0.38–2.02), or sexually transmitted infections (PR = 1.05, 95% CI = 0.62–1.78).

**Table 2:** HBV prevalence and prevalence ratios among pregnant women in Nigeria by sociodemographic characteristics and known risk factors, 2014–2011.

	Number of studies	Number of participants	Number with HBV infection	Pooled prevalence (95% CI)	Prevalence ratio (95% CI)	P-value
Sociodemographic characteristics						
Age						
≥ 25 years	6	4436	160	5.28% (3.13–7.93%)	1.37 (0.89–2.11)	0.158
<25 years	6	1062	26	2.86% (0.85–5.72%)		
Educational attainment						
Secondary or higher	10	3398	330	7.11% (4.30–10.49%)	0.71 (0.58–0.87)	<0.001
None or primary	10	1567	196	6.49% (1.94–12.67%)		
Religion						
Christianity	3	345	28	8.10% (5.38–11.29%)	1.27 (0.65–2.51)	0.483
Islam	3	175	11	6.24% (2.94–10.51%)		
Marital Status						
Married	6	4644	307	5.45% (3.42–7.91%)	0.65 (0.32–1.31)	0.233
Unmarried^a^	6	106	6	1.72% (0.00–9.04%)		
Tribe						
Yoruba	3	239	17	5.59% (2.59–9.37%)	0.79 (0.24–2.60)^b^	0.697
Igbo	3	54	3	2.26% (0.00–10.54%)	1.08 (0.33–3.53)^c^	0.894
Hausa	3	137	18	10.93% (5.49–17.55%)	0.61 (0.20–1.92)^d^	0.401
Risk factors						
Surgery						
Yes	6	1296	130	7.67% (3.65–12.84%)	1.08 (0.90–1.29)	0.409
No	6	5734	536	6.42% (3.12–10.75%)		
Blood transfusion						
Yes	8	652	77	7.11% (2.67–12.99%)	1.19 (0.95–1.48)	0.121
No	8	6802	634	7.53% (4.51–11.23%)		
Multiple lifetime sex partners^e^						
Yes	3	464	22	4.50% (2.71–6.68%)	0.80 (0.35–1.82)	0.603
No	3	726	38	6.79% (2.49–12.83%)		
Tattoos						
Yes	3	448	33	3.99% (0.61–9.14%)	1.02 (0.72–1.45)	0.901
No	3	3172	238	7.38% (6.48–8.33%)		
Tribal marks						
Yes	2	137	0	0.00% (0.00–1.01%)	0.19 (0.02–1.45)	0.108
No	2	245	15	5.73% (3.07–9.08%)		
Scarification						
Yes	3	849	107	5.05% (0.00–19.07%)	0.87 (0.38–2.02)	0.754
No	3	2853	303	8.14% (3.26–14.91%)		
Sexually transmitted infections						
Yes	5	288	15	3.76% (0.98–7.69%)	1.05 (0.62–1.78)	0.851
No	5	1384	76	5.48% (2.72–9.08%)		

^a^ Unmarried includes single and divorced;

^b^Yoruba vs Igbo;

^c^ Igbo vs Hausa;

^d^Yoruba vs Hausa

^e^ Multiple lifetime sex partners: Defined as more than 1 lifetime sexual partner

### Prevalence of HBeAg

**Fig 3:**
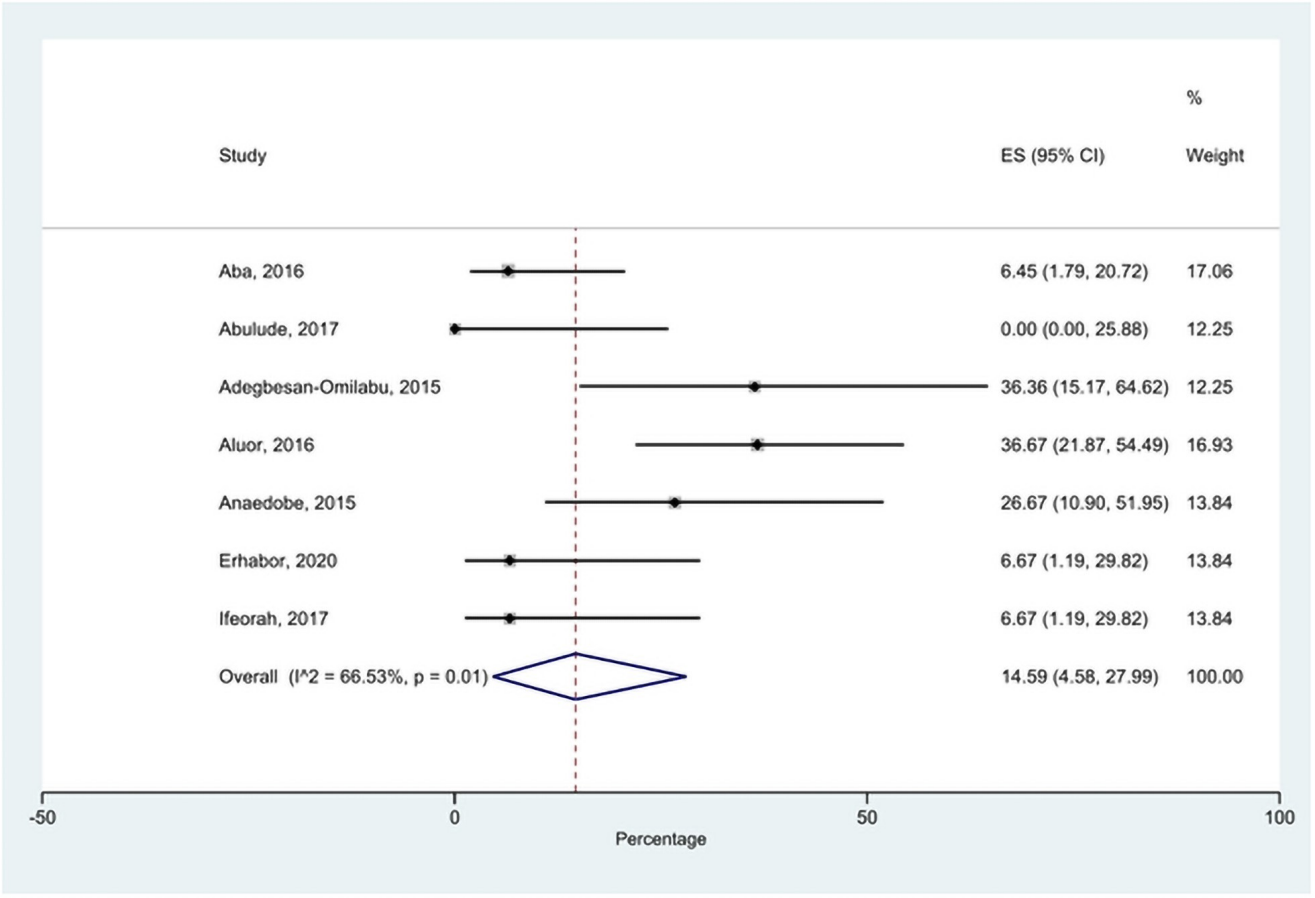
Forest plot of HBeAg prevalence among HBV-infected pregnant women in Nigeria.

Seven studies with a total sample size of 128 reported the prevalence of HBeAg among pregnant women who had HBV infection. The prevalence ranged from 0% to 36.67% (Fig 3). The pooled prevalence of HBeAg across the seven studies was 14.59% (95% CI = 4.58–27.99%, I^2^ = 65.5%, p = 0.01).

### Subgroup analysis

Table 3 (S4 File) shows the subgroup analyses of HBV prevalence based on the region and screening method. The HBV prevalence in the North was 7.61% (95%CI = 5.56–9.95%), while the prevalence in the South was 5.38% (95% CI = 3.84–7.16%) (p-value of difference = 0.104). There was no difference in the prevalence by RDT and ELISA (6.85%; 95% CI = 2.20–13.74%), ELISA only (5.81%; 95% CI = 4.24–7.59%), and RDT only (6.63% (95% CI = 4.59–8.49%) (p-value of difference = 0.808). The findings suggest that the study region and screening method used were not the sources of heterogeneity.

**Table 3:** Subgroup analysis of HBV prevalence among pregnant women in Nigeria, 2014–2021.

Subgroups	Number of studies	Number of participants	Pooled prevalence (95% CI)	I^2^ (p-value)	P-value (subgroup differences)
Region					
North	10	17864	7.61% (5.56–9.95%)	94.7% (<0.001)	0.104
South	10	8684	5.38% (3.84–7.16%)	87.0% (<0.001)	
Screening method					
RDT and ELISA	6	6880	6.85% (2.20–13.74%)	98.6% (<0.001)	0.808
ELISA	6	3433	5.81% (4.24–7.59%)	73.3% (<0.001)	
RDT	8	16235	6.63% (4.59–8.49%)	94.4% (<0.001)	

### Meta-regression

The meta-regression model showed no statistically significant association between the sample size of the studies and HBV prevalence (p = 0.282) (S5 File). Similarly, the association between the study year and HBV prevalence was not statistically significant (p = 0.638) (S5 File). The quality rating of the studies was also not significant (p = 0.470) (S5 File). The findings suggest that none of these variables was the source of heterogeneity.

### Publication bias

**Fig 4:**
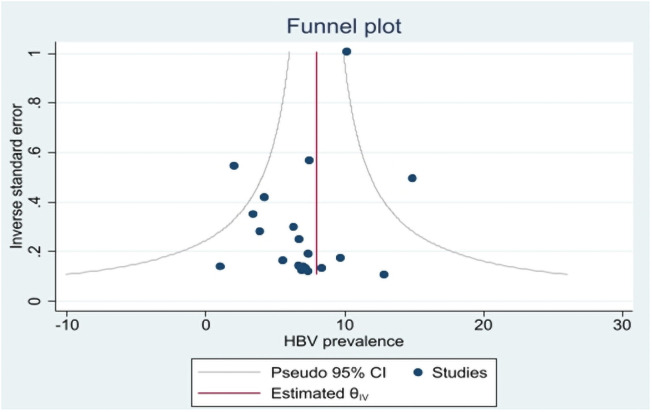
Funnel plot of included studies.

The funnel plot of the studies included in the review suggests no publication bias (Fig 4). The absence of publication bias was further confirmed by the Eggers test (p = 0.778).

## Discussion

To extend the growing body of evidence on HBV in Nigeria, we conducted a systematic review and meta-analysis of the prevalence of HBV among pregnant women using studies published between January 2014 and February 2021. The pooled prevalence of HBV among pregnant women across the 20 studies included in this review was 6.49%. The prevalence of HBV was significantly lower among pregnant women with at least secondary education compared with those with no education or primary education. However, the prevalence of HBV was not significantly different by age, religion, marital status, or tribe. The prevalence of HBV was also not significantly different by known risk factors such as pregnant women with previous surgery, blood transfusion, multiple lifetime sex partners, tribal marks, tattoos, scarification, or sexually transmitted infections. Among HBV-infected pregnant women, the pooled prevalence of HBeAg was 14.59%.

As a result of the expanded HBV vaccination program, the prevalence of HBV infection has decreased, globally but remains highly endemic in some regions, including Africa [61]. Going by the definition of HBV endemicity based on the HBsAg prevalence: low (<2%), lower-intermediate (2–4.99%), higher intermediate (5–7.99%), and high (>8%) [61], our results indicate higher-intermediate endemicity of HBV infection among pregnant women in Nigeria. In line with the World Health Organization (WHO) recommendations [62], Nigeria currently offers a HepB-BD in the national immunization program for children, followed by 3 doses to complete the primary series [24, 63]. HBV vaccination is also recommended for the prevention of HBV in older children, adolescents, and adults, including high-risk populations such as sex workers, medical personnel, and drivers [63]. However, the coverage of HBV vaccines remains suboptimal. The reported estimates of HepB-BD and Hepatitis B 3^rd^ dose (HepB3) coverage in 2019 were 52% and 57%, respectively [64]. Low uptake of HBV vaccines has also been reported among at-risk populations such as health care providers [65, 66]. Several factors, including limited maternal knowledge and unawareness of HBV, unavailability of HBV vaccine, child delivery outside formal health facilities, a long distance from the health facility, and high cost of vaccination, affect the uptake of HBV vaccination in Nigeria [65–69]. Reducing the burden of HBV will require addressing these barriers limiting the vaccination coverage.

The pooled prevalence of HBV in our study compares with the 2018 national household survey in Nigeria that reported a prevalence of 5.9% and among pregnant women [2]. However, it is nearly half of the 14.1% reported in a previous meta-analysis of studies published from 2000–2013 [26]. While the reason for the wide disparity is not clear, this result may suggest a decline in HBV prevalence among pregnant women in Nigeria. Trend studies are needed to examine how the prevalence of HBV infection has changed in Nigeria since the commencement of the vaccination program in 2004. It is noteworthy that the prevalence of HBV was not significantly different by sociodemographic characteristics except educational attainment. Educated women are more likely to be aware of HBV and to have been vaccinated against HBV [70]. This may explain the significant lower prevalence among those who had at least secondary education compared with less-educated women.

Horizontal transmission from infected blood and bodily fluids is a common source of HBV infection in Africa [63, 71–73]. However, in this review, there was insufficient evidence to suggest that the risk factors for horizontal transmission such as previous surgery, blood transfusion, multiple lifetime sex partners, tribal marks, tattoos, scarification, or sexually transmitted infections were associated with HBV among pregnant women in Nigeria. Previous studies have also reported similar results with previous surgery [29, 74], tattooing [29], blood transfusion [29, 74–76], scarification [76], and multiple sex partners [74, 76] among pregnant women. Improvement in blood transfusion safety could have contributed to the reduction in the iatrogenic transmission of HBV infection [77]. Importantly, these findings support a previous recommendation that risk-based HBV screening of pregnant women may not be effective in Nigeria [73]. Household contact is another important source of horizontal transmission that may be responsible for the high burden of HBV among pregnant women [74]. Although the mechanism of household contact transmission of HBV is not fully understood, sharing of personal and household items has been implicated [78–80]. Consequently, contact tracing and screening of household contacts is important in limiting infection spread and should be better incorporated into HBV management protocols in Nigeria.

In this review, we found a high prevalence of HBeAg among HBV-infected pregnant women. Even though HBeAg is not as prevalent in Africa as other high endemic regions [81], it remains a critical risk factor in the perinatal transmission of HBV in the region [12]. Where viral load tests for HBV DNA quantification are not accessible or affordable, HBeAg positive test can be used as a proxy for high HBV DNA among pregnant women [82, 83], which is an indication for additional interventions such as antiviral (tenofovir disoproxil fumarate [TDF]) prophylaxis [82]. TDF prophylaxis, however, is not widely accessible to HBV-infected women in Nigeria, except for those who are co-infected with HIV and may be receiving TDF-based ART through the HIV program [84]. Our findings highlight the need for pregnant women who test positive for HBV in Nigeria to undergo further serological tests to determine their risk of transmission and the appropriate interventions. The availability of reliable and low-cost rapid test kits for HBeAg may improve access to this test in resource-constrained settings [85]. Access to antiviral therapy among HBeAg-positive women should also be prioritized by the government and donor partners.

This review is not without limitations. We considered four databases and might have missed articles in databases not considered. Moreover, many of the studies did not report on the prevalence of HBsAg by sociodemographic characteristics or the prevalence of HBeAg. The differences in the description and categorization of some variables prevented the inclusion of some studies in the meta-analyses. We had planned to assess the difference between HBV prevalence from facility-based and community-based studies. However, we only found one community-based study. More community-based studies are needed on HBV prevalence, considering that many women do not attend health facilities for ANC. Future review studies on the epidemiology of HBV pregnant women in Nigeria should consider extending our findings on the prevalence of HBeAg or assessing HBV DNA levels. Evidence is also required on the rate of perinatal transmission among HBV-infected pregnant women in Nigeria. Although there was an even North-South divide among included studies, future research which focuses on the zones with limited studies may also be warranted.

## Conclusions

There is an intermediate endemicity of HBV infection among pregnant women in Nigeria. As Nigeria continues in its effort to eliminate HBV infection, interventions including routine antenatal HBV screening, antiviral prophylaxis for eligible pregnant women, HBIG, and universal infant vaccination which includes HepB-BD need to be strengthened for the prevention of perinatal transmission of HBV infection.

## Supporting information

S1 File Search strategy for PubMed. (DOCX)

S2 File Data abstraction form and quality assessment tool. (DOCX)

S3 File Forest plots of HBV prevalence ratio by sociodemographic characteristics and risk factors. (DOCX)

S4 File Forest plots of HBV prevalence by study region and screening method. (DOCX)

S5 File Bubble plots of meta-regression of HBV prevalence against sample size, quality rating, and study year. (DOCX)

S1 Checklist (DOCX)
