# Supplementary material for: A systematic review and meta-analysis of the prevalence of hepatitis B virus infection among pregnant women in Nigeria
Source: PLoS One. 2021 Oct 29;16(10):e0259218. doi: 10.1371/journal.pone.0259218 (PMC8555786; doi:10.1371/journal.pone.0259218)
Supplement: S1 File — (DOCX) [file pone.0259218.s001.docx]

Search Strategy for PubMed

“Hepatitis B Virus” OR “HBV” OR “Hep B” OR “Hepatitis B”

AND

 “Pregnant Women” OR “Pregnant” OR “Pregnancy” OR “Antenatal” OR “ANC” OR “Labor” OR “Labour” OR “Delivery”

 AND

 “Nigeria” OR “FCT” OR “Federal Capital Territory” OR “Abuja” OR “Benue” OR “Kogi” OR “Kwara” OR “Nasarawa” OR “Niger” OR “Plateau” OR “Adamawa” OR “Bauchi” OR “Borno” OR “Gombe” OR “Taraba” OR “Yobe” OR “Jigawa” OR “Kaduna” OR “Kano” OR “Katsina” OR “Kebbi” OR “Sokoto” OR “Zamfara” OR “Abia” OR “Anambra” OR “Ebonyi” OR “Enugu” OR “Imo” OR “Akwa Ibom” OR “Bayelsa” OR “Cross River” OR “Delta” OR “Edo” OR “Rivers” OR “Ekiti” OR “Lagos” OR “Ogun” OR “Ondo” OR “Osun” OR “Oyo”

 AND

“Screening” OR “Testing” OR “Prevalence” OR “Positivity” OR “Seropositive” OR “Positive”
