# Supplementary material for: A systematic review and meta-analysis of the prevalence of hepatitis B virus infection among pregnant women in Nigeria
Source: PLoS One. 2021 Oct 29;16(10):e0259218. doi: 10.1371/journal.pone.0259218 (PMC8555786; doi:10.1371/journal.pone.0259218)
Supplement: S2 File — (DOCX) [file pone.0259218.s002.docx]

DATA ABSTRACTION AND QUALITY ASSESSMENT FORM

Reviewer:

Date:

Title of article:

1. First author’s name:
2. Year of publication:
3. Year of study:
4. Type of study:
5. Region of study:
6. Subregion of study:
7. Location of study (state):
8. Sample size:
9. Tests conducted :
10. Method for each test:
11. Number screened for HBsAg:
12. Number positive for HBsAg:
13. Number negative for HBsAg:
14. Number screened for HBeAg:
15. Number positive for HBeAg:
16. Number negative for HBeAg:
17. Sociodemographic factors
18. Age:

|  | Number screened for HBsAg | Number positive for HBsAg | Number negative for HBsAg | Comments |
| --- | --- | --- | --- | --- |
| Category 1 |  |  |  |  |
| Category 2 |  |  |  |  |
| Category 3 |  |  |  |  |
| Category 4 |  |  |  |  |

1. Education

|  | Number screened for HBsAg | Number positive for HBsAg | Number negative for HBsAg | Comments |
| --- | --- | --- | --- | --- |
| Category 1 |  |  |  |  |
| Category 2 |  |  |  |  |
| Category 3 |  |  |  |  |
| Category 4 |  |  |  |  |

1. Religion

|  | Number screened for HBsAg | Number positive for HBsAg | Number negative for HBsAg | Comments |
| --- | --- | --- | --- | --- |
| Category 1 |  |  |  |  |
| Category 2 |  |  |  |  |
| Category 3 |  |  |  |  |
| Category 4 |  |  |  |  |

1. Income

|  | Number screened for HBsAg | Number positive for HBsAg | Number negative for HBsAg | Comments |
| --- | --- | --- | --- | --- |
| Category 1 |  |  |  |  |
| Category 2 |  |  |  |  |
| Category 3 |  |  |  |  |
| Category 4 |  |  |  |  |

1. Others:

|  | Number screened for HBsAg | Number positive for HBsAg | Number negative for HBsAg | Comments |
| --- | --- | --- | --- | --- |
| Category 1 |  |  |  |  |
| Category 2 |  |  |  |  |
| Category 3 |  |  |  |  |
| Category 4 |  |  |  |  |

1. Others:

|  | Number screened for HBsAg | Number positive for HBsAg | Number negative for HBsAg | Comments |
| --- | --- | --- | --- | --- |
| Category 1 |  |  |  |  |
| Category 2 |  |  |  |  |
| Category 3 |  |  |  |  |
| Category 4 |  |  |  |  |

1. Others:

|  | Number screened for HBsAg | Number positive for HBsAg | Number negative for HBsAg | Comments |
| --- | --- | --- | --- | --- |
| Category 1 |  |  |  |  |
| Category 2 |  |  |  |  |
| Category 3 |  |  |  |  |
| Category 4 |  |  |  |  |

1. Risk factors
2. Previous surgery

|  | Number screened for HBsAg | Number positive for HBsAg | Number negative for HBsAg | Comments |
| --- | --- | --- | --- | --- |
| Yes |  |  |  |  |
| No |  |  |  |  |

1. Blood transfusion

|  | Number screened for HBsAg | Number positive for HBsAg | Number negative for HBsAg | Comments |
| --- | --- | --- | --- | --- |
| Yes |  |  |  |  |
| No |  |  |  |  |

1. Multiple sex partners

|  | Number screened for HBsAg | Number positive for HBsAg | Number negative for HBsAg | Comments |
| --- | --- | --- | --- | --- |
| Yes |  |  |  |  |
| No |  |  |  |  |

1. Scarification

|  | Number screened for HBsAg | Number positive for HBsAg | Number negative for HBsAg | Comments |
| --- | --- | --- | --- | --- |
| Yes |  |  |  |  |
| No |  |  |  |  |

1. Tribal marks

|  | Number screened for HBsAg | Number positive for HBsAg | Number negative for HBsAg | Comments |
| --- | --- | --- | --- | --- |
| Yes |  |  |  |  |
| No |  |  |  |  |

1. Others

|  | Number screened for HBsAg | Number positive for HBsAg | Number negative for HBsAg | Comments |
| --- | --- | --- | --- | --- |
| Yes |  |  |  |  |
| No |  |  |  |  |

1. Others

|  | Number screened for HBsAg | Number positive for HBsAg | Number negative for HBsAg | Comments |
| --- | --- | --- | --- | --- |
| Yes |  |  |  |  |
| No |  |  |  |  |

1. Others

|  | Number screened for HBsAg | Number positive for HBsAg | Number negative for HBsAg | Comments |
| --- | --- | --- | --- | --- |
| Yes |  |  |  |  |
| No |  |  |  |  |

Critical Appraisal Checklist

| S/N | Question | Yes: 1; No: O; Unclear; Not applicable | Comments |
| --- | --- | --- | --- |
| 1 | Was the sample frame appropriate to address the target population? |  |  |
| 2 | Were study participants sampled in an appropriate way? |  |  |
| 3 | Was the sample size adequate? |  |  |
| 4 | Were the study subjects and the setting described in detail? |  |  |
| 5 | Was the data analysis conducted with sufficient coverage of the identified sample? |  |  |
| 6 | Were valid methods used for the identification of the condition? |  |  |
| 7 | Was the condition measured in a standard, reliable way for all participants? |  |  |
| 8 | Was there appropriate statistical analysis? |  |  |
| 9 | Was the response rate adequate, and if not, was the low response rate managed appropriately? |  |  |
|  | Total score |  |  |
