# Supplementary material for: A systematic review and meta-analysis of the prevalence of hepatitis B virus infection among pregnant women in Nigeria
Source: PLoS One. 2021 Oct 29;16(10):e0259218. doi: 10.1371/journal.pone.0259218 (PMC8555786; doi:10.1371/journal.pone.0259218)

Forest plots of HBV prevalence ratios by sociodemographic characteristics

1. Age


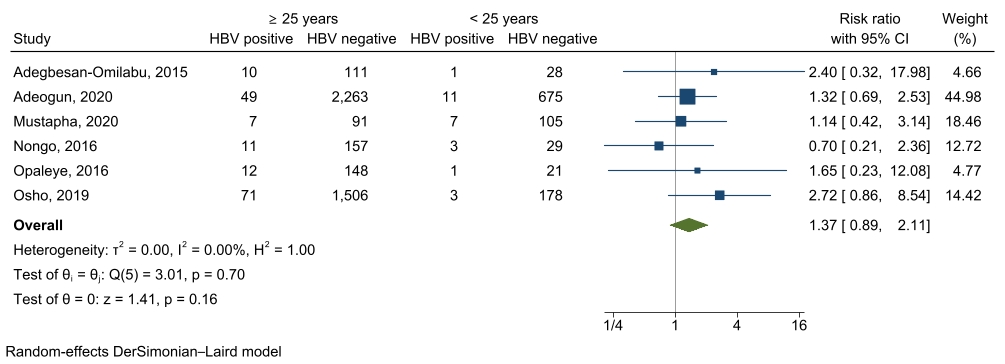


1. Educational attainment


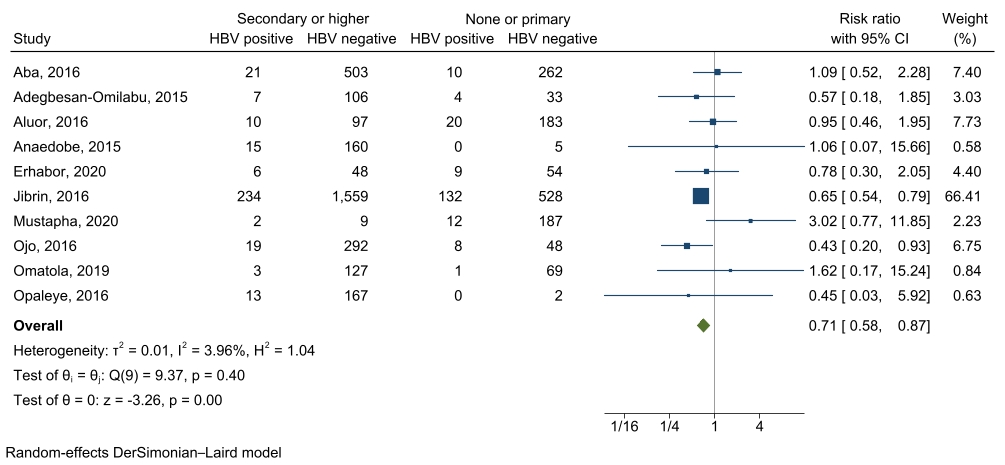


1. Religion


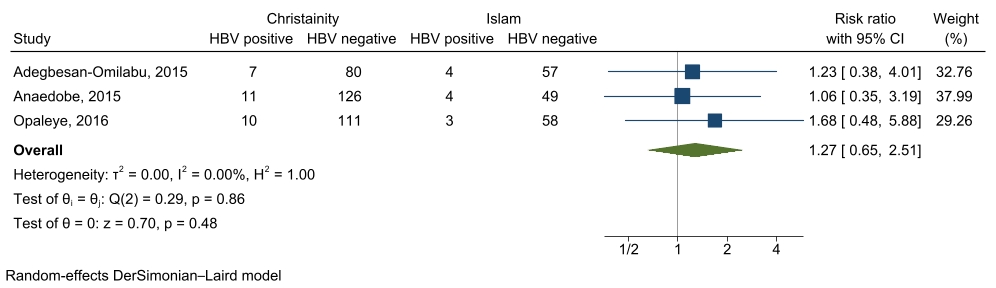


1. Marital status


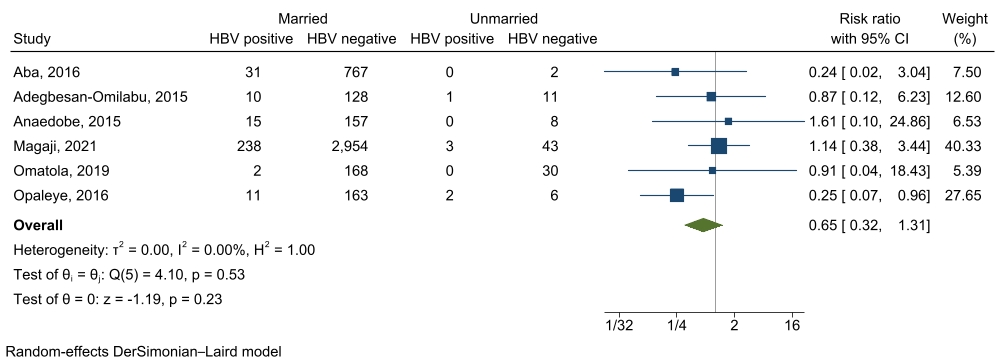


1. Tribe


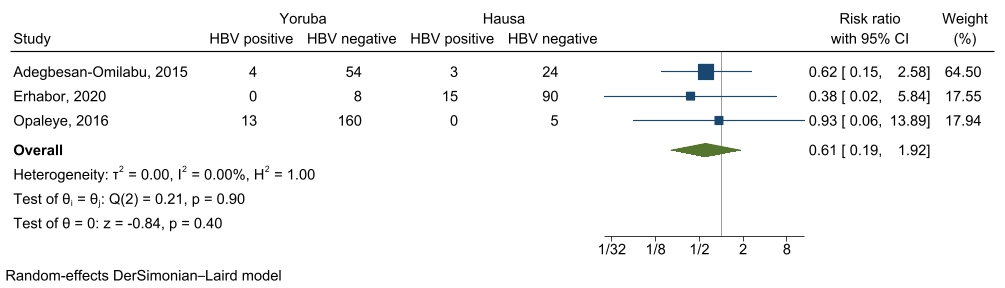


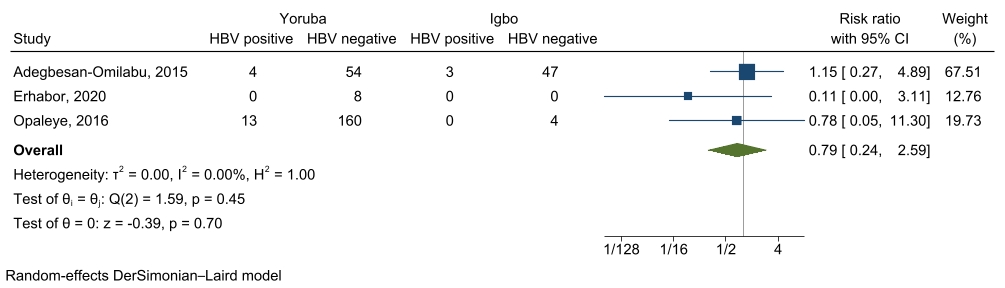


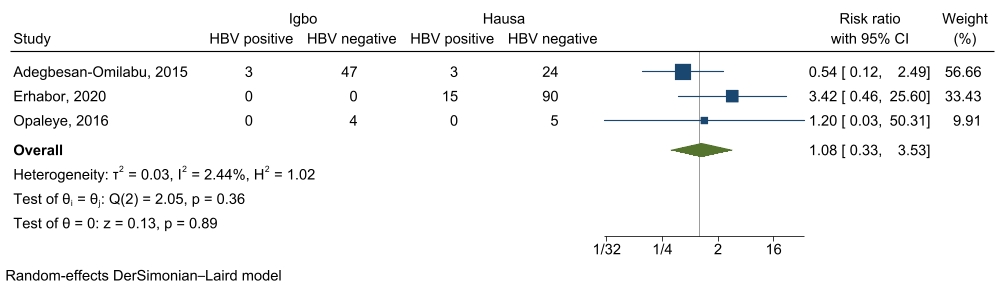


Forest plots of HBV prevalence ratios by risk factors

1. Surgery


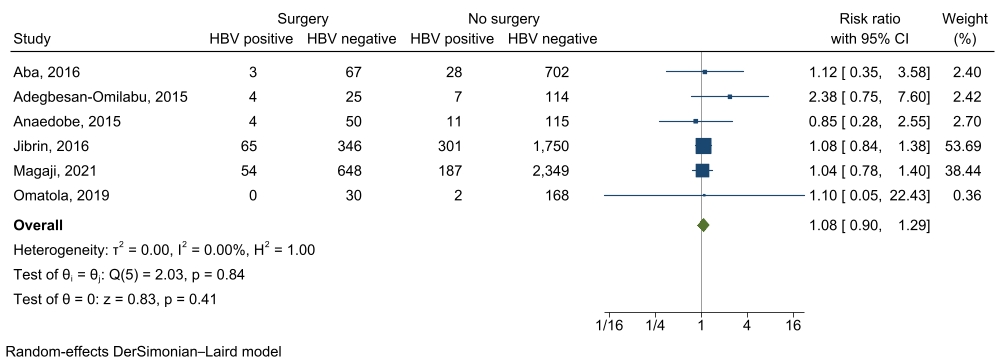


1. Blood transfusion


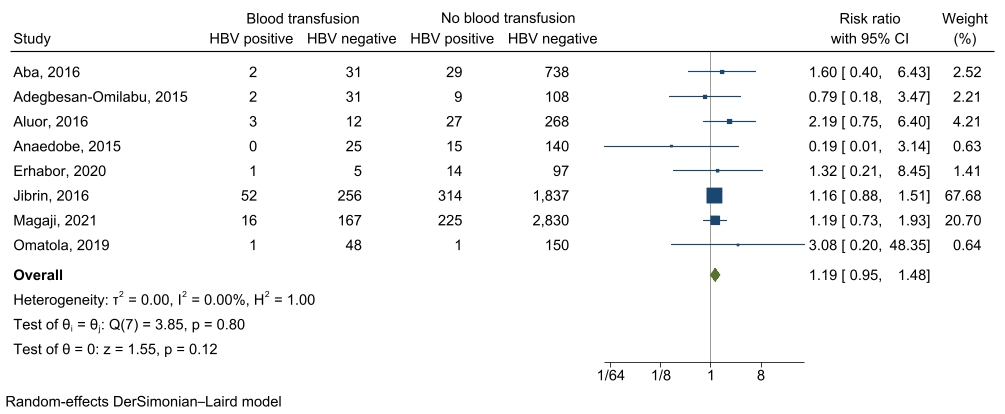


1. Multiple lifetime sex partners


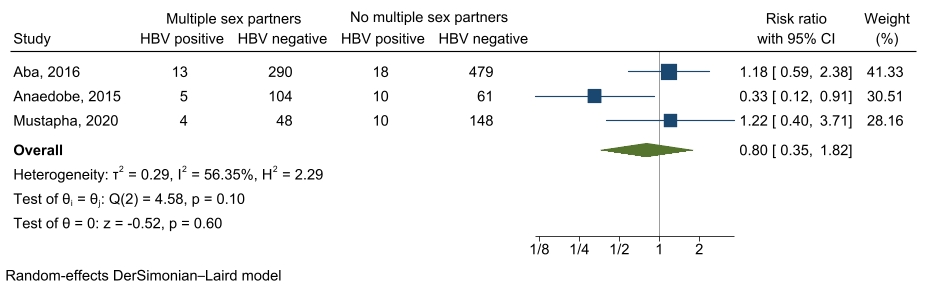


1. Tattoos


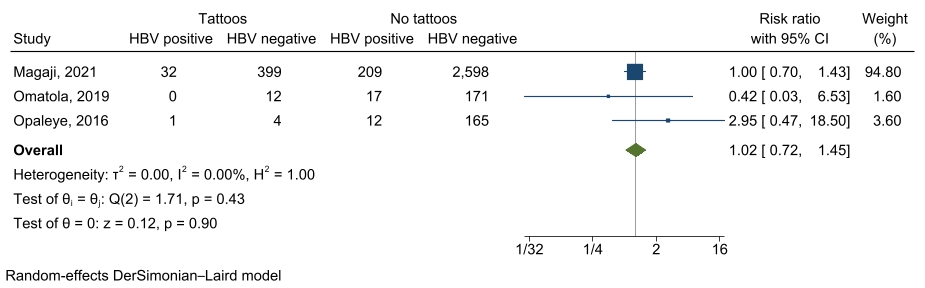


1. Tribal marks


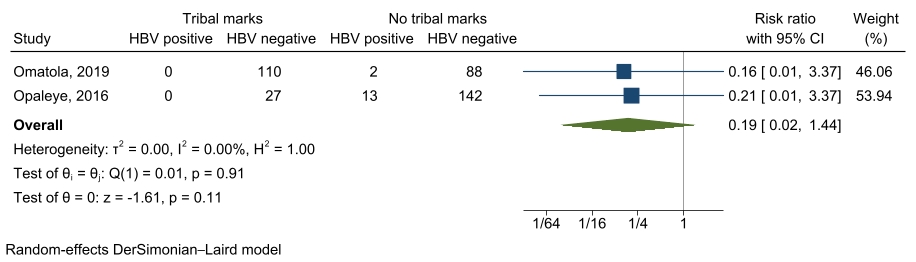


1. Scarification


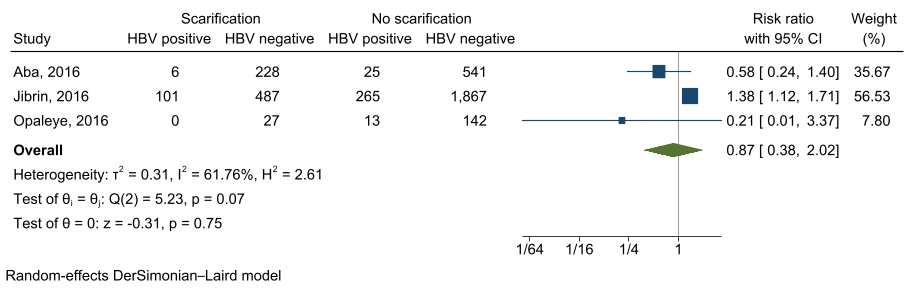


1. Sexually transmitted infections (STIs)


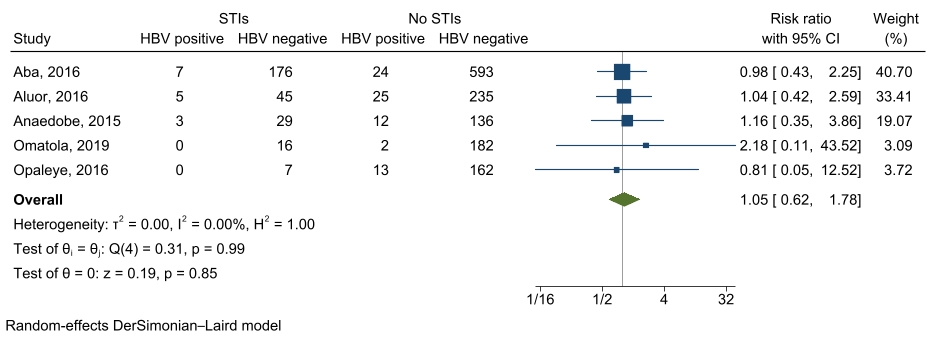

Supplement: S3 File — (DOCX) [file pone.0259218.s003.docx]
