# Supplementary material for: A systematic review and meta-analysis of the prevalence of hepatitis B virus infection among pregnant women in Nigeria
Source: PLoS One. 2021 Oct 29;16(10):e0259218. doi: 10.1371/journal.pone.0259218 (PMC8555786; doi:10.1371/journal.pone.0259218)

1. Forest plot of HBV prevalence among pregnant women by study region


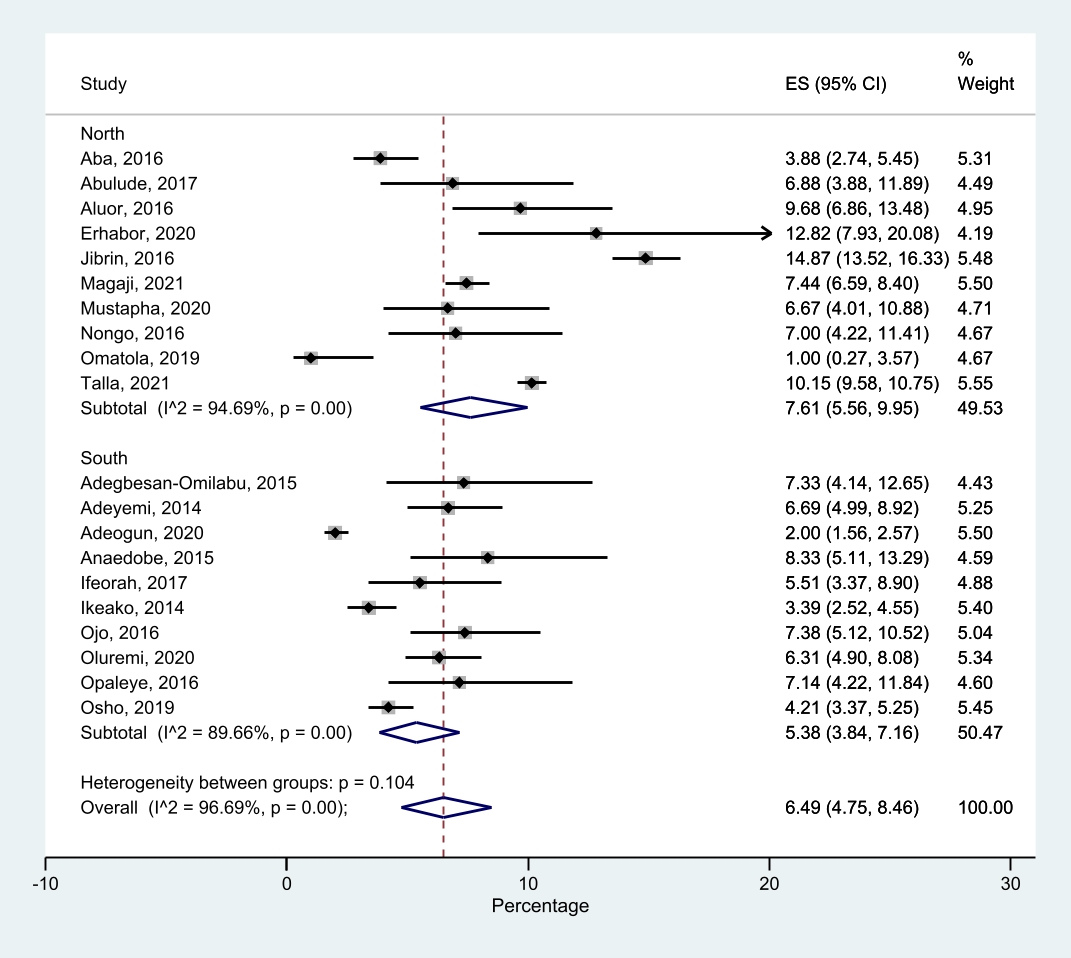


2. Forest plot of HBV prevalence among pregnant women by screening method


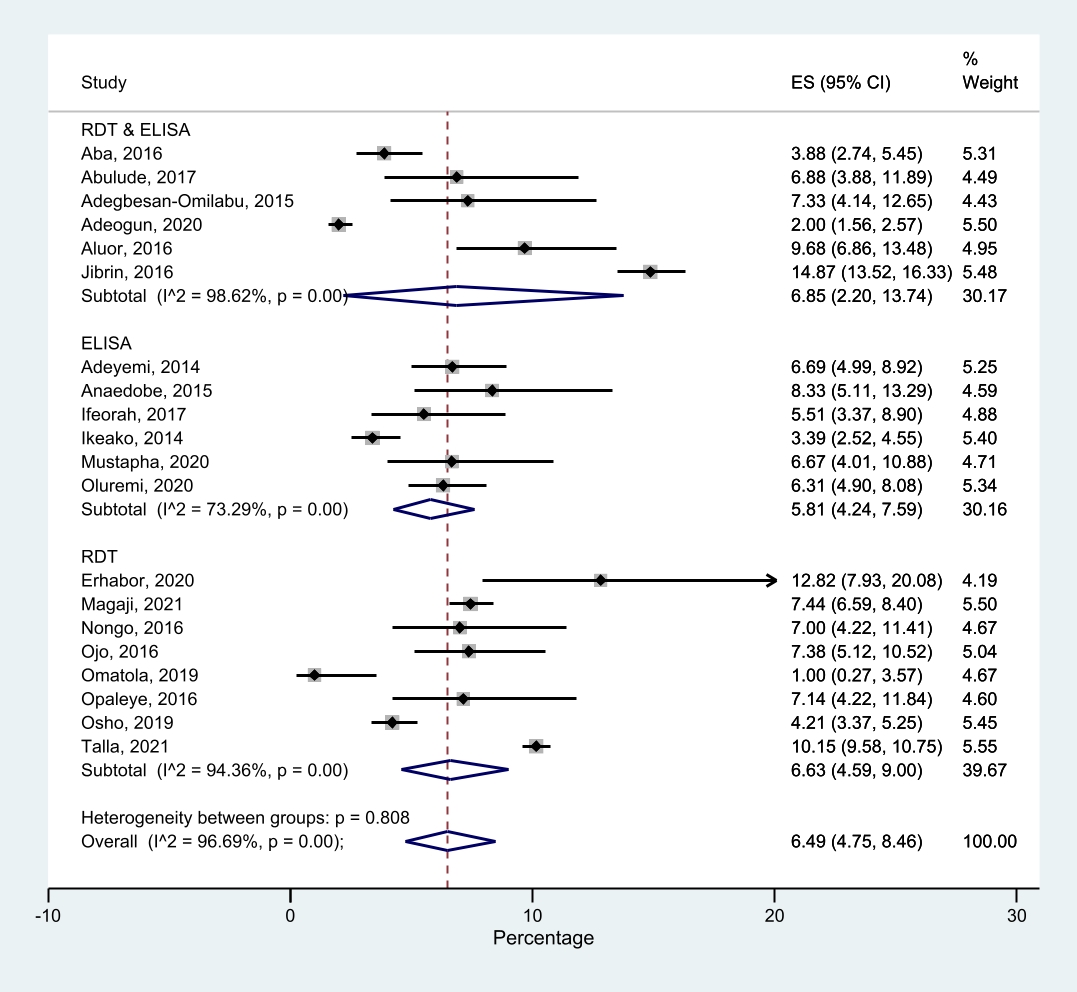

Supplement: S4 File — (DOCX) [file pone.0259218.s004.docx]
