# Supplementary material for: A systematic review and meta-analysis of the prevalence of hepatitis B virus infection among pregnant women in Nigeria
Source: PLoS One. 2021 Oct 29;16(10):e0259218. doi: 10.1371/journal.pone.0259218 (PMC8555786; doi:10.1371/journal.pone.0259218)

1. Meta-regression of HBV prevalence against sample size


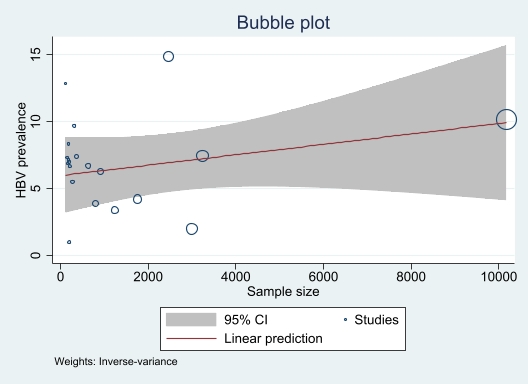


2. Meta-regression of HBV prevalence against quality rating


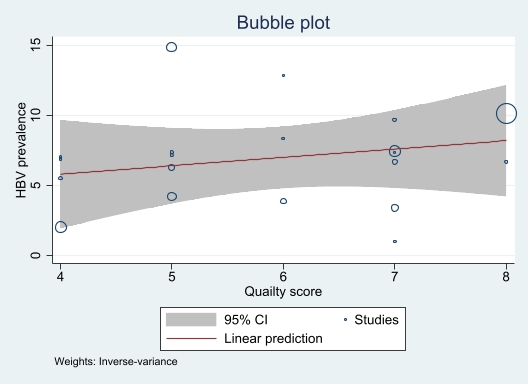


3. Meta-regression of HBV prevalence against study year


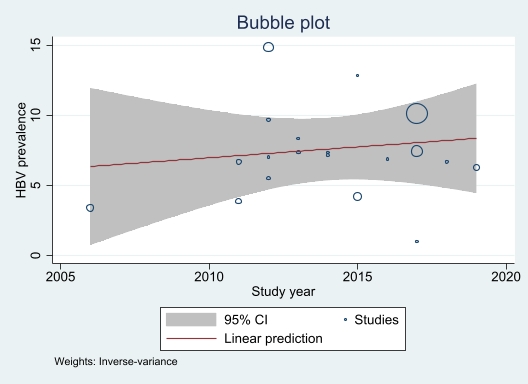

Supplement: S5 File — (DOCX) [file pone.0259218.s005.docx]
